# Mitochondrial DNA Copy Number Variations and Serum Pepsinogen Levels for Risk Assessment in Gastric Cancer

**DOI:** 10.52547/ibj.25.5.323

**Published:** 2021-08-22

**Authors:** Mehdi Alikhani, Samaneh Saberi, Maryam Esmaeili, Valérie Michel, Mohammad Tashakoripour, Afshin Abdirad, Arezoo Aghakhani, Sana Eybpoosh, Massoud Vosough, Mohammad Ali Mohagheghi, Mahmoud Eshagh Hosseini, Eliette Touati, Marjan Mohammadi

**Affiliations:** 1 HPGC Research Group, Medical Biotechnology Department, Biotechnology Research Center, Pasteur Institute of Iran, Tehran, Iran;; 2 Institut Pasteur, Unit of Helicobacter Pathogenesis, CNRS UMR2001, 25-28 Rue du Dr Roux, 75724 Paris Cedex 15, France;; 3 Gastroenterology Department, Amiralam Hospital, Tehran University of Medical Sciences, Tehran, Iran;; 4 Cancer Institute, Tehran University of Medical Sciences, Tehran, Iran;; 5 Clinical Research Dept., Pasteur Institute of Iran, Tehran, Iran;; 6 Department of Epidemiology and Biostatistics, Research Centre for Emerging and Reemerging Infectious Diseases, Pasteur Institute of Iran, Tehran, Iran;; 7 Department of Regenerative Medicine, Cell Science Research Center, Royan Institute for Stem Cell Biology and Technology, ACECR, Tehran, Iran;; 8 Cancer Research Center, Tehran University of Medical Sciences, Tehran, Iran

**Keywords:** Biomarkers, DNA copy number variation, Mitochondrial DNA, Stomach neoplasms

## Abstract

**Background:**

Variations in mtDNA-CN of PBLs, as a potential biomarker for GC screening has currently been subject to controversy. Herein, we have assessed its efficiency in GC screening, in parallel and in combination with sPG I/II ratio, as an established indicator of gastric atrophy.

**Methods:**

The study population included GC (n = 53) and non-GC (n = 207) dyspeptic patients. The non-GC group was histologically categorized into CG (n = 104) and NM (n = 103) subgroups. The MtDNA-CN of PBLs was measured by quantitative real-time PCR. The sPG I and II levels and anti-*H. pylori *serum IgG were measured by ELISA.

**Results:**

The mtDNA-CN was found significantly higher in GC vs*.* non-GC (OR = 3.0; 95% CI = 1.4, 6.4) subjects. Conversely, GC patients had significantly lower sPG I/II ratio than the non-GC (OR = 3.2; CI = 1.4, 7.2) subjects. The combination of these two biomarkers yielded a dramatic amplification of the odds of GC risk in double-positive (high mtDNA-CN-low sPGI/II) subjects, in reference to double-negatives (low mtDNA-CN-high sPGI/II), when assessed against non-GC (OR = 27.1; CI = 5.0, 147.3), CG (OR = 13.1; CI = 2.4, 72.6), or NM (OR = 49.5; CI = 7.9, 311.6) groups.

**Conclusion:**

The combination of these two biomarkers, namely mtDNA-CN in PBLs and serum PG I/II ratio, drastically enhanced the efficiency of GC risk assessment, which calls for further validations.

## INTRODUCTION

Gastric cancer, one of the most mortal cancers worldwide, is detected in more than one million individuals annually and claims an estimated 800,000 lives^[^^1^^]^. This rate is higher in developing countries, including Iran, where GC is the leading cause of cancer death in males^[^^1^^]^. Although the incidence and mortality of GC have shown a slight decrease in recent years, mostly due to the improvements in preventive strategies such as maintaining a healthy and physically active lifestyle^[^^1^^]^, the challenge of timely detection of this silent cancer, for effective therapeutic measures, remains. Thus, identification of efficient biomarkers as candidates for inclusion in blood-based GC screening tests, constitutes a critical area of research^[^^2^^]^. One such strategy can be the quantitation of mtDNA-CN of the PBLs^[^^3^^]^. Mitochondria with a prokaryotic origin play a critical role in the essential functions of eukaryotic cells, including energy production, balancing ROS, autophagy, senescence, and participation in cell signaling pathways^[^^4^^]^.

Mitochondria copy number can be altered in response to different physiological and stress conditions, such as ROS/oxidative stress^[^^5^^]^. Therefore, the observed mtDNA-CN variations in the peripheral blood are considered as a reflection of the above-mentioned factors, mostly owed to the elevation of blood ROS level. Nevertheless, our recent meta-analysis has detected some controversies regarding the efficiency of mtDNA-CN in GC screening^[^^6^^]^.

The available blood-based biomarkers, such as carcinoembryonic antigen and CA19-9^[^^2^^]^, routinely used in clinical practice, have low diagnostic accuracies^[^^7^^]^. Moreover, the diagnostic ability of many other blood-based biomarkers, including circulating tumor cells, certain characteristics of cell-free DNA^[^^8^^]^, various microRNAs, long noncoding RNAs, multiple components of the exosomes, and sPG I/II ratio, remain at research levels^[^^9^^]^. Of these biomarkers, the combination of sPG I and sPG I/II ratio or sPG I/II ratio alone^[^^10^^,^^11^^]^ or plus *H. pylori* serostatus^[^^12^^]^ have long been studied as a noninvasive biomarker for gastric atrophy and cancer screening. 

Herein, we have investigated the ability of mtDNA-CN quantitation, in comparison to, and in combination with serum sPG I/II ratio, in GC risk assessment. Our results indicated that the combination of these two noninvasive blood-based biomarkers substantially amplified the strength of GC risk assessment.

## MATERIALS AND METHODS

**Study population**

Our study population included histologically confirmed GC patients (n = 53), as well as non-GC subjects (n = 207). According to the anatomical location, GC tumors were categorized into cardia, noncardia, or mixed GC subtypes. The non-GC group constituted of nonulcer dyspeptic patients, with CG (n = 104) or NM (n = 103). Non-GC patients with the histologic grades of inflammation and atrophy stages of 0-I and those with II-IV were considered as NM and CG, respectively. We primarily compared GC patients against the non-GC group and then took a step further to compare every two groups (i.e. GC vs*.* CG, GC vs*.* NM, and CG vs*.* NM). 

**Interview data and sample collection**

Using a structured questionnaire, participants were asked for their demographic characteristics, including age, gender, ethnicity (Fars or non-Fars), smoking status (never or ever), and FHGC, in their first-degree relatives (yes/no). Five milliliters of fasting whole blood were taken from each participant, half of which was used for serum collection, and the other half for PBL isolation and DNA extraction. Tissue samples were obtained under gastroscopy or gastric surgery, fixed in formalin, and stained with hematoxylin and eosin. The stained slides were analyzed, by expert pathologists, in a blinded fashion. 

**Gastric histopathology**

According to the modified Sydney system^[^^13^^]^, the following gastric specimens were obtained from: (1) the anterior and posterior walls of the proximal corpus (C1–C2 = oxyntic mucosa), (2) the greater and lesser curvatures of the distal antrum (A1–A2 = mucus-secreting mucosa), and (3) the lesser curvature at the incisura angularis (I). The grades (0-IV) of inflammation and stages (0-IV) of atrophy were determined based on the OLGA method of classification^[^^14^^,^^15^^]^. The grading and staging of gastric tumors were carried out using the TNM (T: primary tumor, N: regional lymph nodes, M: distant metastasis) system^[^^16^^]^. Tumor subtypes were identified as intestinal, diffuse, signet ring cell, or mixed^[^^17^^]^.

***H. pylori*** **serostatus**

*H. pylori*-specific IgG antibodies were detected by ELISA assays (Serion ELISA Classic, Germany), following the manufacturers’ protocols. *H. pylor*i-positive and -negative serum samples were recognized accordingly, and those with borderline titers were repeated. 

**PBL DNA extraction**

The DNA was extracted from the whole blood using the salting-out method^[^^18^^]^.

**MtDNA-CN quantification**

Quantification of mtDNA-CN was conducted as previously described^[^^19^^]^, using a real-time PCR system (Applied Biosystem 7500, Thermofisher, USA). The 12S ribosomal RNA gene, which is specific for mitochondrial DNA, was amplified using forward and reverse primers as follows: F: 5´-GCTCGCCAG AACACTACGAG-3’; R: 5´-CAGGGTTTGCTGAAG ATGGCG-3’. For the quantitation of nuclear DNA, 18S rRNA, which is specific to nuclear DNA, the following primers were used: F: 5´-GAGAAACGGC TACCACATCC-3’and R: 5´-GCCTCGAAAGAGTC CTGTAT- 3’. Each reaction contained 5 μl of DNA (40 pg/µl), 0.2 μl of each primer (10 μmol/L), 10 μl of SYBR green master mix, and 4.8 μl of double-distilled water, making a total volume of 20 μl. The amplification reaction was as follows: initial denaturation at 95 ºC for 10 minutes, followed by 40 cycles of 95 ºC for 15s 60 ºC for 1 min, and finally a dissociation step for drawing the melting curve. Samples were analyzed in triplicates. The relative mtDNA-CN was measured using the ΔC_t_ of average C_t_ of mtDNA and nuclear DNA [ΔCt = CT_nuclear DNA-CT_mtDNA], as 2^ ΔCt^.

**sPG I and II measurement**

Levels of sPG I and II were measured by ELISA assay (BIOHIT, Finland) based on the manufacturer’s protocol. The ratio of sPG I to sPG II (sPG I/II) was calculated and reported. 

**Statistical analyses**

The demographic and clinicopathologic characteristics were described for GC, non-GC, CG, and NM groups. All analyses were carried out twice, once comparing GC vs*. *non-GC cases and again comparing the three groups, i.e., GC, CG, and NM. The normality of the quantitative variables was assessed by the Kolmogorov Smirnov test, and the non-parametric method was used for all statistical analyses, due to skewness of biomarker data. The 95% CI for the proportions was estimated using the Clopper-Pearson method^[^^20^^]^. Pearson Chi-Square test was used to assess the association between clinical groups and demographic and clinicopathological features. In each study group, mean mtDNA-CN was compared between the above-mentioned categories using the Mann-Whitney U test. MtDNA-CN was also compared between the categories of tumor subsite (cardia/noncardia/mixed), tumor stage (IA/IB/II/ IIIA/IIIB/IV), tumor grade (un-differentiated / poorly differentiated/moderately differentiated/well-differentiated), and tumor type (intestinal/diffuse/signet ring cell/mixed), using the Kruskal Wallis test. The same approach was followed in order to compare the mean sPGI/II ratio in the above-mentioned clinicopathologic categories. ROC curve analysis was performed to estimate the AUC and the performance of mtDNA-CN and sPGI/II ratio in discriminating GC from non-GC cases, as well as GC from CG and NM groups. ROC curve analysis was also used to identify optimum cut-off values, based on the highest sensitivity and specificity rates. To determine the OR for mtDNA-CN and sPGI/II ratio, patients in each group were divided into low and high groups, categorized based on their cut-off values. The sPG and mtDNA-CN were then combined, and patients were labeled as one of the four possible outcomes. The ORs were calculated using multinomial regression analysis, both in crude (without adjusting for the demographic variables) and adjusted (for age, gender, ethnicity, smoking, *H. pylori* serostatus, and FHGC) formats. Statistical tests were considered significant at 0.05 levels. Data were analyzed using SPSS statistics software (version 24) and Graphpad prism (version 8).

**Ethical statement**

The above-mentioned sampling protocols were approved by the Ethics Committee of Pasteur Institute of Iran, Tehran (ethical code: IR.PII.REC.1394.57). Every participant provided a written informed consent, before undergoing interview for their demographic and lifestyle factors, as well as blood and gastric sampling.

## RESULTS

**The demographic and clinicopathologic characteristics of study participants**

Our patient population consisted of 260 subjects, amongst whom 53 were diagnosed with histologically confirmed gastric adenocarcinoma (GC) and the rest (n = 207) with nonulcer dyspepsia, who are herein referred to as non-GC patients. Of the latter group, 104 subjects had some degrees of gastritis and the rest (n = 103) presented with histologically NM. The gastric tumors of the GC patients were mostly located in the noncardia region (71%) and were of the intestinal subtype (51%). GC patients were significantly older than non-GC subjects (59 ± 1 vs*.* 52 ± 1; *p* < 0.001; Table 1). Amongst the non-GC group, CG patients were older than those with NM (54 ± 1 vs*.* 49 ± 1; *p* = 0.004). GC patients were mainly male (74%), with non-Fars ethnicity (85%, Table 1). Most of the GC cases (67%) were *H. pylori*-seropositive, slightly higher but similar to non-GC subjects (57%), amongst whom the CG patients had a significantly higher *H. pylori *seropositivity (71%) than those with NM (42%, *p* < 0.001; Table 1). The majority of GC cases (56%) were ever smokers (*p* = 0.001; Table 1). The mtDNA-CN and sPG I/II ratio were primarily analyzed in various demographic and clinicopathologic subgroups (Table 2). These analyses revealed that GC patients over the age of 60 years had significantly higher mtDNA levels (6.1 ± 3.8 vs. 4.1 ± 2.9; *p* = 0.031). The same was true for GC patients with FHGC (7.7 ± 4.8 vs*.* 4.5 ± 3.0; *p* = 0.017). The average sPGI/II ratio was lower in non-GC subjects, older than 60 (8.4 ± 4.5 vs. 10.4 ± 5.5; *p* = 0.030). In the CG group, this ratio was lower in *H. pylori*-seropositive individuals compared to seronegatives (8.8 ± 5.1 vs*.* 11.3 ± 5.3; *p* = 0.003). Therefore, age, gender, ethnicity, smoking status, and FHGC were considered as potential confounding variables for adjustment, when assessing the ORs. 

**Table 1:** The demographic and clinicopathologic characteristics of study participants

**Variables**	**Number (%)**		***p*** ** value**	**Number (%)**		***p *** **value**		
	**GC** **(n = 53)**	**Non-GC** **(n = 207)**	**GC vs** ***.*** **non-GC**	**CG** **(n = 104)**	**NM** **(n = 103)**	**GC vs** ***.*** **CG**	**GC vs** ***.*** **NM**	**CG vs** ***.*** ** NM**
**Age** (mean ± SD)	59 ± 12	52 ± 12	**0.001**	54 ± 12	49 ± 12	**0.010**	**<0.001**	**0.004**
**Age category**								
< 60	32 (60)	157 (76)		73 (71)	84 (82)			
** ≥ **60	21 (40)	49 (24)	**0.021**	30 (29)	19 (18)	0.214	**0.003**	0.072
**Gender**								
Female	14 (26)	104 (50)		56 (54)	48 (47)			
Male	39 (74)	103 (50)	**0.002**	48 (46)	55 (53)	**0.002**	**0.010**	0.297
**Ethnicity**								
Fars	8 (15)	89 (43)		47 (46)	42 (41)			
Non-Fars	44 (85)	117 (57)	**0.001**	56 (54)	61 (59)	**<0.001**	**0.001**	0.482
***H. pylori *** **sero-status**								
Negative	17 (33)	89 (43)		29 (28)	60 (58)			
Positive	35 (67)	116 (57)	0.289	74 (72)	42 (42)	0.680	**0.010**	**<0.001**
**Smoking**								
Never	23 (44)	144 (70)		77 (75)	67 (65)			
Ever	29 (56)	62 (30)	**0.001**	26 (25)	36 (35)	**<0.001**	**0.010**	0.181
**FHGC**								
No	40 (83)	179 (90)		89 (90)	90 (91)			
Yes	8 (17)	19 (10)	0.160	10 (10)	9 (9)	0.275	0.164	0.809
**Inflammation grade**								
0	-	37 (18)		0 (0)	37 (36)			
I	-	71 (34)		5 (5)	66 (64)			
II	-	72 (35)		72 (69)	0			
III	-	13 (6)		13 (13)	0			
IV	-	14 (7)	-	14 (13)	0	**-**	**-**	**<0.001**
**Atrophy stage**								
0	-	148 (72)		51 (49)	97 (94)			
I	-	7 (3)		1 (1)	6 (6)			
II	-	34 (16)		34 (33)	0			
III	-	17 (8)		17 (16)	0			
IV	-	1 (1)	-	1 (1)	0	**-**	**-**	**<0.001**
**Tumor subsite**								
Cardia	12 (24)			-	-			
Noncardia	36 (71)	-		-	-			
Mixed	2 (4)	-		-	-			
**Tumor stage**								
IA	1 (2)	-		-	-			
IB	6 (13)	-		-	-			
II	11 (23)	-		-	-			
IIIA	9 (19)	-		-	-			
IIIB	12 (26)	-		-	-			
IV	8 (17)	-		-	-			
**Tumor grade**								
Undifferentiated	1 (2)	-		-	-			
Poorly differentiated	19 (41)	-		-	-			
Moderately differentiated	17 (37)	-		-	-			
Well differentiated	9 (20)	-		-	-			
**Tumor subtype**								
Intestinal	18 (51)	-		-	-			
Diffuse	10 (29)	-		-	-			
Singet ring cell	1 (3)	-		-	-			
Mixed	6 (17)	-		-	-			

**Table 2:** Distribution of mtDNA and sPG I/II ratio in various demographic and clinicopathologic subgroups

**Variables**	**mtDNA-CN (Mean ** **± SD)**				**sPG I/II** **(Mean ** **± SD)**			
	**GC**	**Non-GC**	**CG**	**NM**	**GC**	**Non-GC**	**Gastritis**	**NM**
**Age**								
< 60	4.1 ± 2.9	2.9 ± 2.4	3.6 ± 2.8	2.4 ± 1.8	5.9 ± 4.7	10.4 ± 5.5	9.1 ± 4.4	11.3 ± 6.2
≥ 60	6.1 ± 3.8	2.7 ± 2.1	3.2 ± 2.2	2.0 ± 1.9	5.8 ± 2.9	8.4 ± 4.5	7.1 ± 4.7	10.4 ± 3.5
***p*** ** value**	**0.031**	0.643	0.500	0.459	0.939	**0.030**	**0.048**	0.517
**Gender**								
Female	3.7 ± 3.2	2.9 ± 2.0	3.6 ± 2.1	2.2 ± 1.7	4.5 ± 3.2	10.3 ± 5.5	8.8 ± 4.3	11.8 ± 6.2
Male	5.3 ± 3.5	2.8 ± 2.5	3.3 ± 3.0	2.4 ± 1.9	6.2 ± 4.1	9.5 ± 5.1	8.2 ± 4.8	10.5 ± 5.3
***p*** ** value**	0.146	0.737	0.563	0.582	0.261	0.310	0.493	0.267
**Ethnicity**								
Fars	4.7 ± 2.6	2.8 ± 2.1	3.3 ± 2.3	2.2 ± 1.7	3.3 ± 1.9	9.5 ± 5.2	8.5 ± 4.5	10.4 ± 5.8
Non-Fars	5.0 ± 3.6	2.9 ± 2.4	3.5 ± 2.8	2.4 ± 1.9	6.2 ± 4.0	10.2 ± 5.4	8.5 ± 4.6	11.7 ± 5.7
***p*** ** value**	0.777	0.709	0.699	0.681	0.152	0.319	0.958	0.270
***H.*** ***pylori*** ** serology**								
Negative	4.7 ± 3.6	2.8 ± 2.2	3.6 ± 2.4	2.5 ± 1.9	4.8 ± 3.6	11.3 ± 5.3	9.7 ± 5.0	12.1 ± 5.4
Positive	5.0 ± 3.4	2.9 ± 2.4	3.4 ± 2.7	2.0 ± 1.6	5.1 ± 3.4	8.8 ± 5.1	7.2 ± 3.8	10.0 ± 6.2
***p*** ** value**	0.707	0.970	0.918	0.471	0.707	**0.003**	0.217	0.131
**Smoking**								
Never	4.7 ± 3.3	2.8 ± 2.2	3.3 ± 2.4	2.2 ± 1.7	6.1 ± 3.4	9.9 ± 5.4	8.3 ± 4.2	11.5 ± 5.9
Ever	5.2 ± 3.5	3.0 ± 2.6	3.8 ± 3.1	2.5 ± 2.0	5.9 ± 3.4	9.9 ± 5.3	9.2 ± 5.5	10.3 ± 5.4
***p*** ** value**	0.644	0.496	0.454	0.443	0.865	0.992	0.421	0.288
**FHGC**								
No	4.5 ± 3.0	2.9 ± 2.4	3.5 ± 2.7	2.3 ± 1.8	5.4 ± 3.8	9.9 ± 5.5	8.4 ± 4.6	11.3 ± 5.9
Yes	7.7 ± 4.8	2.8 ± 1.9	3.2 ± 1.8	2.4 ± 2.1	6.2 ± 2.3	10 ± 4.5	8.9 ± 4.5	11.3 ± 5.4
***p*** ** value**	**0.017**	0.949	0.745	0.826	0.586	0.930	0.765	0.980
**Inflammation grade**								
0	-	1.8 ± 1.3	-	1.9 ± 1.3	-	11.1 ± 6.2	-	11.1 ± 6.2
I	-	2.5 ± 2.0	1.1 ± 0.8	2.6 ± 2.0	-	10.8 ± 4.9	9.4 ± 6.1	10.8 ± 4.9
II	-	3.8 ± 2.6	3.9 ± 2.6	-	-	8.2 ± 5.0	8.3 ± 5.1	-
III	-	2.3 ± 1.6	2.3 ± 1.6	-	-	8.7 ± 2.3	8.7 ± 2.3	-
IV	-	3.0 ± 2.8	3.0 ± 2.8	-	-	12.1 ± 6.5	11.3 ± 6.9	-
***p*** ** value**	-	**0.0001**	**0.029**	**0.05**	**-**	**0.007**	**0.315**	0.761
**Atrophy grade**								
0	-	2.9 ± 2.2	4.2 ± 2.4	2.3 ± 1.8	-	10.1 ± 5.4	8.5 ± 5.1	10.9 ± 5.5
I	-	2.3 ± 2.1	1.8 ± 0	2.4 ± 2.3	-	10.8 ± 3.5	5.9 ± 0	11.6 ± 3.0
II	-	2.5 ± 2.5	2.5 ± 2.5	-	-	9.1 ± 5.7	8.9 ± 5.8	-
III	-	2.9 ± 2.9	2.9 ± 2.9	-	-	9.4 ± 4.4	9.4 ± 4.4	-
IV	-	3.2 ± 0	3.2 ± 0	-	-	-	-	-
***P*** ** value**	-	0.833	**0.042**	0.894	-	0.734	0.881	0.758
**Tumor subsite**								
Cardia	4.6 ± 3.1	-	-	-	4.1 ± 2.9	-	-	-
Non-cardia	4.7 ± 3.3	-	-	-	6.5 ± 4.2	-	-	-
Mixed	4.1 ± 3.9	-	-	-	2.4 ± 0	-	-	-
***p*** ** value**	**0.020**	-	-	-	0.320	-	-	-
**Tumor stage**								
IA	7.8 ± 0	-	-	-	5.3 ± 0	-	-	-
IB	3.4 ± 1.9	-	-	-	3.7 ± 2	-	-	-
II	7.1 ± 3.7	-	-	-	5.4 ± 2.8	-	-	-
IIIA	5.8 ± 4.2	-	-	-	7.7 ± 4.4	-	-	-
IIIB	3.4 ± 1.7	-	-	-	5.9 ± 5.0	-	-	-
IV	3.8 ± 1.9	-	-	-	6.2 ± 4.9	-	-	-
***p*** ** value**	**0.034**	-	-	-	0.683	-	-	-
**Tumor grade**								
Undifferentiated	5.0 ± 0	-	-	-	11.3 ± 0	-	-	-
Poorly differentiated	3.7 ± 2.1	-	-	-	4.4 ± 4.8	-	-	-
Moderately differentiated	6.1 ± 4.5	-	-	-	5.6 ± 3.2	-	-	-
Well differentiated	5.8 ± 3.2	-	-	-	5.9 ± 3.9	-	-	-
***p*** ** value**	0.168	-	-	-	0.595	-	-	-
**Tumor type**								
Intestinal	5.3 ± 3.3	-	-	-	5.5 ± 3.2	-	-	-
Diffuse	3.4 ± 2.8	-	-	-	5.7 ± 5.8	-	-	-
Signet ring cell	2.0 ± 0	-	-	-	3.2 ± 0	-	-	-
Mixed	5.1 ± 1.7	-	-	-	2.6 ± 1.3	-	-	-
***P *** **value**	0.320	-	-	-	0.454	-	-	-

**Fig. 1:**
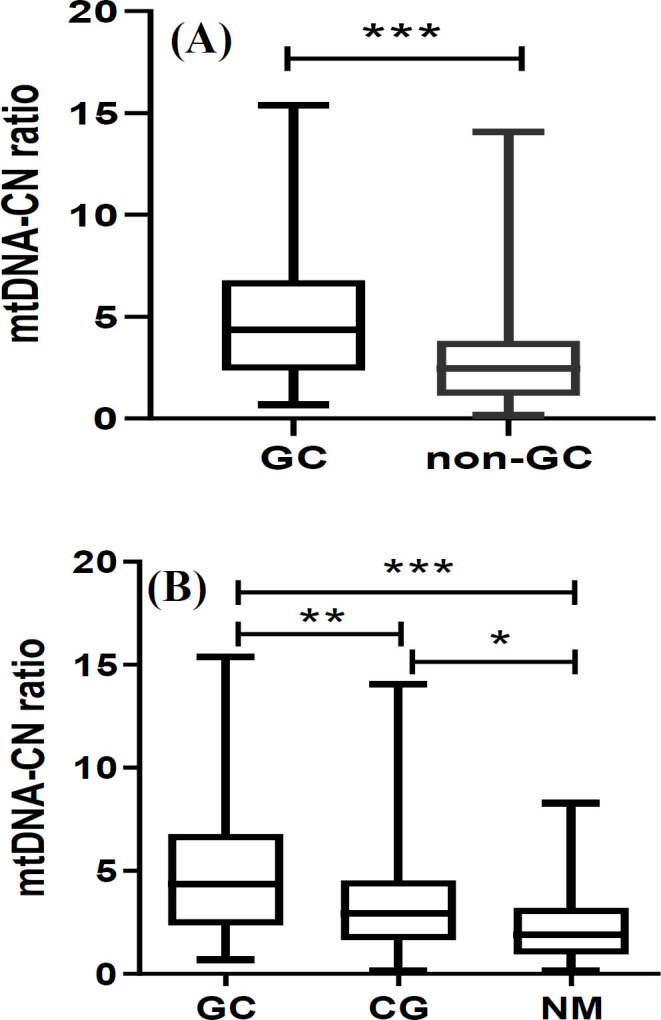
mtDNA-CN. (A) GC vs*.* non-GC and (B) amongst the subgroups. ^*^, ^**^ and ^***^ represent statistical significance of <0.05, <0.01 and <0.001, respectively

**MtDNA-CN of PBLs**

The mtDNA-CN in GC patients was significantly higher than the non-GC subjects (4.9 ± 3.4 vs*.* 2.8 ± 2.3; *p* = 0.001; Fig. 1A). The higher copy number was mainly contributed by the difference observed between GC and NM subjects (2.3 ± 1.8; *p *< 0.001) and to a lesser extent by CG patients (3.4 ± 2.6; *p* = 0.01; Fig. 1B). Again, mtDNA-CN was slightly higher in CG patients as compared to subjects with NM (2.3 ± 1.8; *p* = 0.04, Fig. 1B). ROC curve analysis showed statistically significant diagnostic performances for mtDNA-CN, in discriminating GC from non-GC group, and its subgroups (AUC = 0.64-0.77; Table 3). To assess the risk impact of this biomarker, being associated with each clinical diagnosis, the crude and adjusted ORs (for age, gender, ethnicity, smoking, *H. pylori*, and FHGC) for every two groups were calculated (Table 4). Subjects with high mtDNA content were at threefold increased risk of GC compared to non-GC (OR_adjusted _= 3.0; *p* = 0.004; Table 4A). This risk was further amplified when GC patients were compared to NM (OR_adjusted_ = 5.2; *p* < 0.001; Table 4C). In addition, those with high mtDNA content were at higher risk of CG, when compared to NM subjects (OR_adjusted_ = 2.7; *p* = 0.004; Table 4D).

**sPG I/II ratio**

**Fig. 2:**
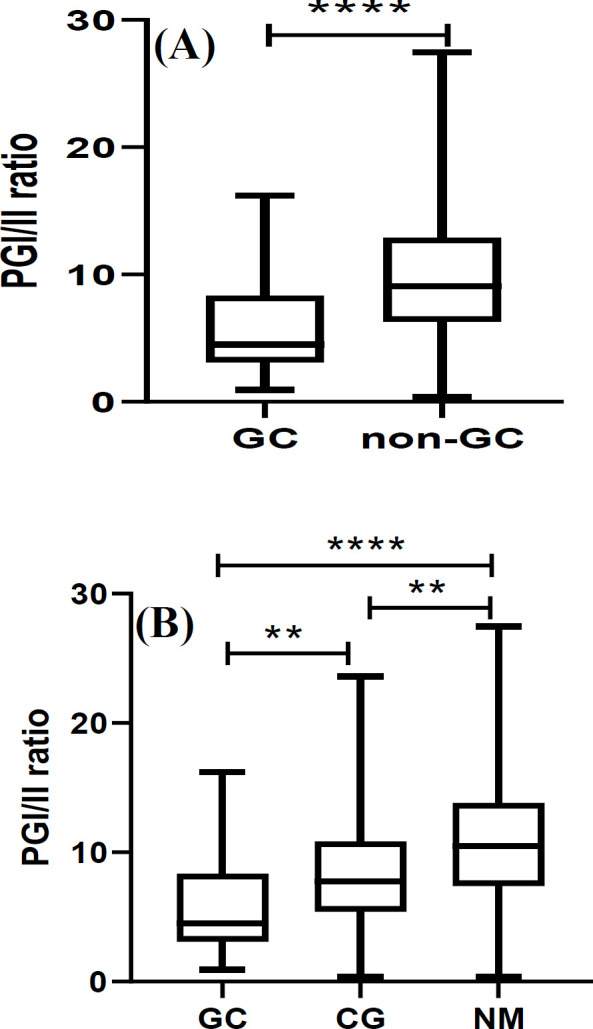
Serum PGI/II ratio. (A) GC vs*.* non-GC and (B) amongst the subgroups. ^**^ and ^***^ represent statistical significance of <0.01 and <0.001, respectively

Contrary to mtDNA-CN, the mean sPG I/II ratio of GC patients was significantly lower than that of the non-GC subjects (5.8 ± 3.9 vs*.* 9.8 ± 5.4; *p* < 0.001; Fig. 2A). This was owed mostly to the difference observed between GC and NM subjects (10.9 ± 5.4; *p* < 0.001; Fig. 2B) and to a lesser extent to CG patients (8.5 ± 4.5; *p* = 0.01; Fig. 2B), for whom sPG I/II ratio was also lower than NM subjects (*p* = 0.01; Fig. 2B). ROC curve analysis showed good ability for sPG I/II ratios in discriminating GC from non-GC and its subgroups (AUC = 0.65-0.79), as shown in Table 3. 

**Table 3:** ROC curve for the screening performance of mtDNA-CN and sPGI/II

	**AUC**	**SE**	**95% CI**	***p*** ** value**	**Cut-off**	**Sensitivity (%)**	**Specificity (%)**
**mtDNA-CN**							
GC vs*.* non-GC	0.71	0.04	0.63, 0.78	<0.001	3.33	64	70
GC vs*.* CG	0.64	0.05	0.55, 0.74	0.003			
GC vs*.* NM	0.77	0.04	0.69, 0.85	<0.001	2.92	72	72
CG vs*.* NM	0.65	0.04	0.57, 0.72	<0.001			
**PGI/II**							
GC vs*.* non-GC	0.74	0.43	0.65, 0.82	<0.001	7.55	63	66
GC vs*.* CG	0.68	0.05	0.58, 0.79	0.001			
GC vs*.* NM	0.79	0.04	0.70, 0.87	<0.001	8.16	66	70
CG vs*.* NM	0.65	0.04	0.57, 72	<0.001			

**Table 4:** Single and double assessment of mtDNA-CN and sPG I/II ratio in risk screening

	Risk variables					OR (95% CI)			
A	mtDNA	sPGI/II		GC	Non-GC	Crude	*p* value	Adjusted	*p* value
Single	low	-		19	143	Ref	-	Ref	
	high	-		34	64	4.0 (2.1, 7.5)	< 0.001	**3.0 (1.4, 6.4)**	0.004
	-	high		16	130	Ref	-	Ref	-
	-	low		28	74	3.1 (1.6, 6.1)	0.001	**3.2 (1.4, 7.2)**	0.006
Double	low	high		4	89	Ref	-	Ref	-
	low	low		10	51	4.4 (1.3 to 14.6)	0.017	**7.4 (1.6, 33.9)**	0.011
	high	high		14	41	7.6 (2.4 to 24.5)	0.001	**8.0 (1.9, 34.2)**	0.002
	high	low		16	22	15.5 (4.7, 50.8)	< 0.001	**27.1 (5.0, 147.3)**	< 0.001
									
B	**mtDNA**	**sPGI/II**		**GC**	**CG**	**Crude**	***p*** ** value**	**Adjusted**	***p*** ** value**
Single	low	-		15	53	Ref	-	Ref	-
	high	-		38	51	2.5 (1.2, 5.2)	0.011	2.0 (0.9, 4.5)	0.114
	-	high		13	48	Ref	-	Ref	-
	-	low		31	54	2.1 (1.0, 4.5)	0.053	2.3 (0.9, 5.6)	0.076
Double	low	high		4	34	Ref	-	Ref	-
	low	low		10	28	3.0 (0.9, 10.7)	0.085	**5.6 (1.2, 27.0)**	0.033
	high	high		14	32	5.4 (1.6, 18.6)	0.006	**6.8 (1.4, 32.9)**	0.017
	high	low		16	18	7.6 (2.2, 26.0)	0.001	**13.1 (2.4, 72.6)**	0.003
									
C	**mtDNA**	**sPGI/II**		**GC**	**NM**	**Crude**	***p*** ** value**	**Adjusted**	***p*** ** value**
Single	low	-		15	73	Ref	-	Ref	-
	high	-		38	29	6.6 (3.1, 13.7)	<0.001	**5.2 (2.2, 12.3)**	<0.001
	-	high		13	68	Ref	-	Ref	-
	-	low		31	34	4.7 (2.2, 10.1)	<0.001	**3.8 (1.5, 9.7)**	0.004
Double	low	high		4	55	Ref	-	Ref	-
	low	low		10	23	6.0 (1.7, 21.0)	0.005	**8.5 (1.8, 40.5)**	0.007
	high	high		14	19	10.1 (3.0, 34.6)	<0.001	**11.7 (2.3, 58.4)**	0.003
	high	low		16	5	44.0 (10.6, 183.5)	<0.001	**49.5 (7.9, 311.6)**	<0.001
									
D	**mtDNA**	**sPGI/II**		**CG**	**NM**	**Crude**	***p*** ** value**	**Adjusted**	***p*** ** value**
Single	low	-		53	73	Ref	-	Ref	-
	high	-		51	29	2.6 (1.5, 4.6)	0.001	**2.7 (1.4, 5.2)**	0.004
	-	high		48	68	Ref	-	Ref	-
	-	low		54	34	2.2 (1.3, 3.9)	0.006	1.7 (0.9, 3.2)	0.104
Double	low	high		34	55	Ref	-	Ref	-
	low	low		28	23	2..0 (1.0, 4.0)	0.057	1.5 (0.7, 3.4)	0.303
	high	high		32	19	1.9 (0.9, 4.0)	0.100	1.7 (0.7, 4.0)	0.219
	high	low		18	5	5.8 (2.0, 17.1)	0.001	**3.8 (0.9, 15.3)**	0.063

*Adjusted for age, gender, ethnicity, *H. pylori *serostatus, smoking, and FHGC. OR, odds ratio; CI, confidence interval

Subjects with low sPG I/II ratios were at more than threefold increased risk of GC, compared to non-GC (OR_adjusted_ = 3.2; *p* = 0.006; Table 4A). The measured risk of GC vs*.* non-GC was mostly contributed by the difference between GC vs*.* NM (OR_adjusted_ = 3.8; *p* = 0.004; Table 4C). 

**Joint assessment of mtDNA-CN and sPG I/II ratio**

Joint assessment of mtDNA-CN-sPG I/II ratio produced the following four groups: (1) low-high (reference group), (2) low-low, (3) high-high, and (4) high-low. The crude and adjusted ORs for GC risk sequentially increased from 7.4 to 27.1 fold for groups 2 to 4, respectively, in comparison to the reference group (Group 1; Table 4A). In every comparison, double positive subjects (Group 4) were identified at the highest risk, as compared to the other groups, including GC vs*.* non-GC (OR_adjusted_ = 27.1; *p* < 0.001; Table 4A), GC vs*.* CG (OR_adjusted_ = 13.1; *p* = 0.003; Table 4B), GC vs*.* NM (OR_adjusted_ = 49.5; *p* < 0.001; Table 4C), and finally CG vs*.* NM (OR_adjusted_ = 3.8; ***p*** = 0.063; Table 4D) groups.

## DISCUSSION

Our study is the first to report the combination efficacy of mtDNA-CN and sPGI/II for discriminating GC from non-GC (CG and NM) patients. Results showed an elevation of mtDNA-CN in GC compared 

to non-GC and also in CG in comparison with NM. Our data support the potential of the mtDNA-CN assay as a noninvasive biomarker for GC screening, especially when combined with sPGI/II.

In different types of cancer, changes in PBL mtDNA have emerged as a potent noninvasive biomarker associated with cancer risk^[^^21^^]^. In this study, variations in mtDNA content of PBLs were evaluated as a biomarker for gastric tissue alterations, including tumor and gastritis. The fact that PBL mtDNA can reflect tissue changes is not yet clear, but there are many hypotheses for this process. For instance, an elevated blood ROS level has been proposed as a cause^[^^19^^,^^22^^]^. This elevation can in turn lead to perturbations in the function of mitochondria^[^^23^^]^ or activation of phagocytic leukocytes^[^^24^^]^, which may ultimately affect the mtDNA-CN in blood leukocytes^[^^25^^]^. Other participating factors, such as activated immune cells^[^^26^^,^^27^^]^ and genetic defects/alterations affecting mitochondrial genome^[^^28^^]^, as well as demographic factors, such as age^[^^29^^]^ and smoking^[^^30^^]^, may be involved in mtDNA-CN variations. In the present report, a significant increase was observed in mtDNA-CN in GC patients aged over 60 years. On the other hand, a substantial increase in the mtDNA-CN of gastritis patients, compared to the NM group, implies the participation of activated leukocytes as proposed previously^[^^26^^,^^27^^]^. Moreover, this elevation was divergent with varying grades of inflammation. 

Our data, with the ability of mtDNA-CN in dissociating GC from non-GC patients, are consistent with previous reports on Mexican^[^^19^^]^ and Chinese patients^[^^26^^]^. Also, compared to other cancers of the gastrointestinal tract, our data are in line with reports on Indian^[^^31^^]^ and Chinese CRC^[^^32^^]^, as well as American oral cancer patients^[^^33^^]^. On the contrary, there are studies that have reported lower mtDNA-CN in the PBLs of gastric^[^^34^^]^ and esophageal^[^^35^^]^ cancers. Observing no significant differences in mtDNA-CN between healthy and cancer patients has also been reported in CRC^[^^36^^]^ and GC^[^^37^^,^^38^^]^. These discrepancies could be due to various confounding factors, such as the stage of cancer, which we have discussed in our recent meta-analysis on the mtDNA-CN in gastrointestinal cancers^[^^6^^]^. Overall, the meta-analysis results showed higher mtDNA-CN in gastrointestinal cancers, as reported in case-control studies. On the contrary, the mtDNA-CN was found to be lower in patients prior to cancer development, as reported in nested case-control studies^[^^37^^,^^39^^,^^40^^]^. Consistently, lower mtDNA levels were observed in the gastric mucosa of mouse models, which developed chronic inflammation and preneoplastic lesions, such as dysplasia^[^^41^^]^. Intriguingly, in the present study, the Spearman’s correlation test indicated a negative association between mtDNA-CN and stage of atrophy in CG patients (R^2 ^= -0.364; *p *< 0.001). These data suggest that mtDNA-CN may be elevated in gastritis, suppressed prior to cancer establishment, and again raised following cancer initiation. However, the exact mechanisms of this dynamic change during gastric carcinogenesis remain unclear. 

Our analysis indicated that there are variations amongst the DNA extraction methods, used. Amongst these approaches, the use of spin column-based nucleic acid purification kits, remains the method of choice. The second most frequently used methods included the phenol-chloroform and salting-out methods^[^^6^^]^. Gue *et al.*^ [^^42^^]^ compared two silica-based column kits, with the phenol-chloroform-isoamyl alcohol extraction methods^[^^42^^]^. They demonstrated that the former method had the least degree of intra-sample variation. These authors also reported loss of mtDNA, following serial passage of genomic DNA through the kits' columns. Their results emphasize taking into account the type of DNA extraction method, when evaluating the resultant mtDNA-CN variations. Another source of variation can be the sequence of primers, used for nuclear and mitochondrial amplification. Choosing appropriate primers for this purpose can profoundly impact the outcome of mtDNA quantitation^[^^5^^]^.

sPGI/PGII ratio is also considered as a valuable noninvasive biomarker for the detection of gastric atrophy, as a preneoplastic lesion^[^^43^^]^. Our data showed decreased sPGI/II levels in GC compared to non-GC, and its power of discrimination *via* this biomarker was consistent with previous reports, as summarized by Huang *et al.*^[^^44^^]^. The sPG I and II logically reflect the status of gastric mucosa, as they are secreted by the chief cells of gastric glands^[^^45^^]^. Although the reported efficacy of sPG for GC screening are substantial, some discrepancies are observed^[^^45^^]^.

We have previously reported the added value of sPG in combination with *H*. *pylori* for GC screening^[^^12^^]^. In the present study, we have probed the effectiveness of the combination of sPG with mtDNA-CN. This combination led to substantially higher ORs in discriminating GC from non-GC subjects. Considering the improvement in the performance was highly remarkable, this strategy may be promising as a complementary test for sPGI/II measurement. Nonetheless, because of the wide CI, mostly due to the relatively small sample sizes in each group, these findings need to be confirmed in larger populations. 

The combined efficiency of biomarkers has formerly been reported for GC screening. For instance, Sasazuki et al.^[^^46^^]^ have combined the *H. pylori* infection status with CagA and sPG levels, which more than doubled the efficiency of GC risk estimation. In another study, sPG status was combined with barium digital radiography, which yielded a highly effective GC screening, in different GC subgroups^[^^47^^]^. The combination of sPG levels with miR-101-3p^[^^48^^]^ and high sensitive C-reactive protein^[^^49^^]^ also showed promising results for distinguishing between atrophic gastritis and GC and increasing the sensitivity of GC screening from 61% to 73%.

The strengths of our study include the sub-stratification of patients according to endoscopic and histologic observations. Furthermore, every analysis was carefully adjusted for multiple potentially confounding demographic factors. The limitations, however, include the small sample sizes, in the combined risk groups. 

The combination of high mtDNA-CN and low sPG I/II ratio enhanced the efficiency of GC, as well as CG,risk assessment, substantially. Our study provides the lead for further investigations and validation of joint assessment of mtDNA-CN and sPG I/II ratio in larger studies, including subjects of various geographic origins, which could potentially result in the development of high performance, yet noninvasive, GC screening tools.
